# An Overview of Ontologies in Virtual Reality-Based Training for Healthcare Domain

**DOI:** 10.3389/fmed.2021.698855

**Published:** 2021-07-09

**Authors:** Ummul Hanan Mohamad, Mohammad Nazir Ahmad, Youcef Benferdia, Azrulhizam Shapi'i, Mohd Yazid Bajuri

**Affiliations:** ^1^Institute of IR4.0, Universiti Kebangsaan Malaysia, Bangi, Malaysia; ^2^Faculty of Information Science & Technology, Universiti Kebangsaan Malaysia, Bangi, Malaysia; ^3^Department of Orthopaedics and Traumatology, Faculty of Medicine, Universiti Kebangsaan Malaysia, Kuala Lumpur, Malaysia

**Keywords:** knowledge representation, ontology engineering, domain knowledge, virtual reality, medical education and training

## Abstract

Virtual reality (VR) is one of the state-of-the-art technological applications in the healthcare domain. One major aspect of VR applications in this domain includes virtual reality-based training (VRT), which simplifies the complicated visualization process of diagnosis, treatment, disease analysis, and prevention. However, not much is known on how well the domain knowledge is shared and considered in the development of VRT applications. A pertinent mechanism, known as ontology, has acted as an enabler toward making the domain knowledge more explicit. Hence, this paper presents an overview to reveal the basic concepts and explores the extent to which ontologies are used in VRT development for medical education and training in the healthcare domain. From this overview, a base of knowledge for VRT development is proposed to initiate a comprehensive strategy in creating an effective ontology design for VRT applications in the healthcare domain.

## Introduction

Virtual reality is a technology advancement that creates an immersive, virtual environment to allow users to interact and visualize the real world in a virtual form (1) through multiple sensory channels (2). As mentioned by Ajmera and Gonen (3), VR is commonly composed of three main components; art, audio and mechanics. The first component, art, describes the environment where users see, interact with objects and the animated surroundings in VR. Audio, on the other hand, provides some level of immersiveness and reality-like feeling during the real-time simulation of the VR. Meanwhile, mechanics is the main part of the VR that facilitates user interaction with the created virtual events.

In the healthcare domain, VR-based training often involves the use of VR headsets, instrumented clothing such as haptic gloves or tracking suits, along medical instruments. So far, a few VRTs in the healthcare domain have been developed to provide an alternative channel for medical education and training for healthcare personnel. Among them are virtual surgery for ophthalmology, laparoscopic, endoscopic procedures (4), anatomy dissection (5), emergency simulation (6), and many other procedural trainings (7). Hence, the need and scope for VRT in the healthcare domain are limitless. VRT in the healthcare domain is an interactive, immersive use of VR technology for medical education and training purposes, to provide a real-time simulation of an actual setting (8) related to the healthcare domain. One of the perks of having VRT in the healthcare domain is that it provides a progressive way to train healthcare personnel in a safe, controlled environment (9) while reducing the potential risks that exist when the training is performed on the actual patients (10). However, the development of VRT in its current state is time-consuming and complicated due to certain limitations such as accessibility, cost of virtual tools, perception of the VR technology and usability (11). Elaboration on these limitations under the ontological perspective will be further discussed in section Literature Review. Moreover, the knowledge from developing one VRT in the healthcare domain is rarely used to speed up the development of another VRT (12). Hence, there is a need to tackle this limitation to expand VRT development in the healthcare domain by understanding how the knowledge on this can be shared and reused across other VRT training activities.

One of the well-known mechanisms suitable for capturing knowledge and making it explicit for seamless sharing of information in a domain of interest is ontology (13, 14). Some studies have investigated the use of ontologies in the intersection of Ontology Engineering and virtual reality (15). However, the use of ontologies, specifically VRT, is not well-captured and is missing all the aspects and evidence that we are interested in. For example, how ontology has been used in VRT to manage the learning scenario, users' behavior, and interaction inside VR is not something that has been distinctly established. Therefore, this paper represents the field of ontology and gives an overview of the recent research in the field, in the context of medical education and training. The research questions of this paper are focused on finding the existing types of ontologies, the methodologies for building ontologies and defining the purpose of these ontologies for VRT in healthcare.

**Figure 1:**
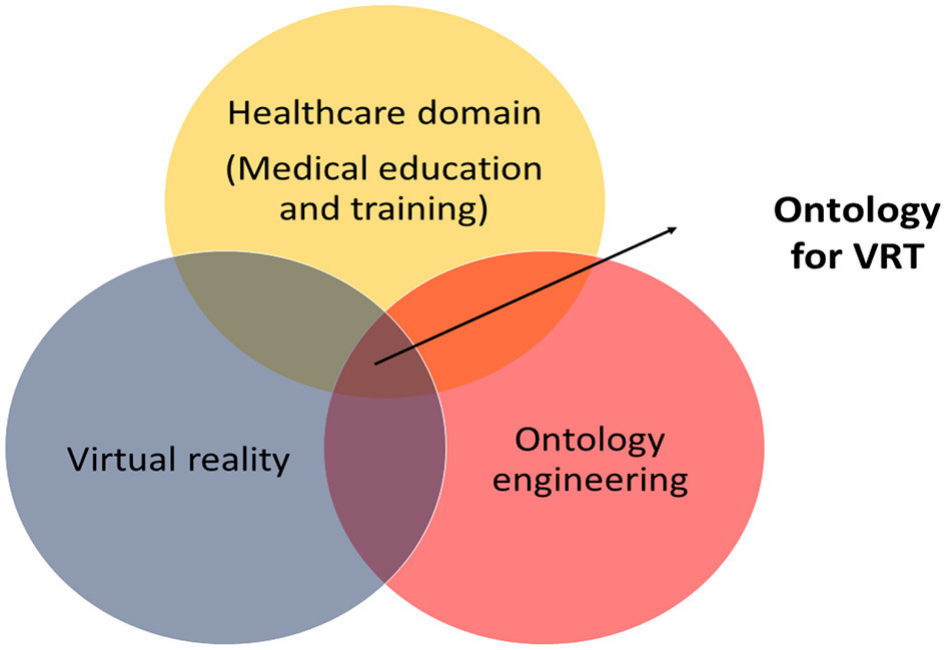
The scope of ontology review in VRT.

Figure 1 depicts the scope of this study. By demonstrating this, the focus is illuminated toward understanding the ontologies that currently exist to support VRT in medical education and training, what are the missing elements in the existing ontologies and what is yet to be explored in VRT in the healthcare domain from an ontological perspective.

The rest of the paper is structured as follows. Section Literature Review includes the general applications of VRT, issues in VRT development, ontology classification and the role of ontology in VRT development based on the context of healthcare. In section Methodology, we present the existing ontologies developed for VRT in the healthcare domain, providing an ontological perspective to be discussed in section Ontologies Application in VRT in Healthcare Domain. Then, section Discussion concludes the work with potential future directions.

## Literature Review

In general, Zhou et al. (16) mentioned that the role of VR in medical education and training can be classified into two types; VR as a teaching tool or VR as a learning environment. VR as a teaching tool can be defined as “a visualization tool that uses VR technologies to engaging users to learn spatially complex topics” (17). Meanwhile, VR as a learning environment simulates complex objects in virtual form to facilitate better understanding and visualization.

### General Applications of Virtual Reality in Healthcare

The virtual reality-based applications in the field of healthcare are growing tremendously with the advancement in technology, especially now, in the era of the fourth industrial revolution. In general, these applications fall into the following categories.

#### Virtual Surgery

VR simulations are now progressively being used for numerous surgical practices such as in ophthalmology, laparoscopic, endoscopic, and even cataract surgery procedures. The main purpose is to allow surgeons, regardless of their expertise level, to rehearse and practice complex surgical procedures using virtual reality before attempting the procedures on a patient. Previously, these types of training exercises were often performed during real surgery through observations and then, under the supervision of senior surgeons (18). Hence, when there are good training alternatives such as through VRT, this opens bigger opportunities for surgeons to enhance their surgery skills, with less need to practice on real patients. With realistic haptic feedback, surgeons can assess their performance. In agreement, a study by Thomsen et al. (19) revealed that the operating room performance improves when surgeons undergo VRT training.

#### Anatomy Dissection

Another essential virtual reality-based training in healthcare is anatomy dissection. Using VR, the anatomy of important organs such as the ear, bone, and others can be visualized and explored up to the level of intricate details that will help facilitate accurate dissection skills among surgeons. According to Jang et al. (5), VR-based training is more sustainable compared to human cadavers in long term. To accentuate the enormous potential of using VR in anatomy dissection, virtual training is now shaped to allow active manipulation of the 3D structures rather than just passive viewing. To add, VRT in anatomy dissection has reported a significant increase in the overall confidence among surgeons post-VRT training (20).

#### Disease Management

The use of virtual reality is also expanded to training in disease management. One important example is the VRT to train healthcare professionals that work with patients suffering from mental diseases. As mental diseases are not the type that impacts patients physically, it can be quite challenging to diagnose and provide accurate treatments and interventions. Through VR, physicians can experience what happens inside the mind of a patient with schizophrenia (21). The possibility of having VRT for this purpose eventually will carve a bigger path for the expansion of many VRT developments in the healthcare domain.

#### Emergency Simulation

A medical emergency is one of the most important training types for any healthcare professional. Hence, it only seems fitting that virtual reality technology is also adopted to develop VRT for emergency simulation. This type of training is important to ensure that healthcare personnel can respond immediately and effectively during any medical crisis. Using VR, simulations of any probable events can also be created. McGrath et al. (6) highlighted that having VR training for emergency simulations is beneficial in a way that it provides an environment that overcomes the issues of limited clinical training hours while allowing trainers to focus on improving their skills and training.

#### Procedural Trainings

Virtual reality is also applied in healthcare for procedural and communication training (21). To add, this becomes more important especially during the pandemic crisis of COVID-19 disease outbreak, when it is not possible to perform training in normal ways. Procedural training using VR may include the common medical standard operating procedures (SOPs), clinician-patient communication and more. A study by Sowndararajan et al. (22) mentioned that the immersiveness capability of VR training resulted in better compliance of the healthcare personnel toward the procedures. This is because they can efficiently remember complex procedures. In other words, VRT for procedural training can facilitate healthcare practitioner competencies and minimize unnecessary errors.

### Limitations in Developing VRT in Healthcare

The healthcare domain is complex. Hence, implementing VR for medical education and training is quite a trivial task. Many adopters in the healthcare field have encountered problems at different phases within VRT development.

Firstly, as in many other domains, the development of VRT in healthcare is often limited by the difficulty to maintain a standardized vocabulary (23). This happens often due to the involvement of stakeholders of varying competencies and skills. For example, both subject matter experts (SMEs) and technological developers have their understanding of the central concepts. As a result, the same word may mean different things in different contexts or different words used in different domains could mean the same thing (24). This gap contributes toward complicating the development of VRTs for the healthcare domain (25). Until now, VRT remains expensive, complicated and time-consuming (26).

Secondly, there is a high rate of failure in VRT development in the healthcare domain (27). One of the reasons behind this is the lack of adequate information exchange and communication that supports the whole development process. So far, there are no clear and comprehensive guidelines on how to develop VRT for healthcare. Additionally, the VRT knowledge areas are also not explored in-depth. Although there is a basic workflow on VR development, many VRTs in healthcare are developed in-silo, for the certain specialization of skills and therefore, are non-reusable for the development of another VRT within the same domain (28).

Thirdly, the healthcare domain is a field rich in terminologies (29). Yet, the rapid accumulation of these various terminologies, taxonomies, tools, and applications has led to a more complicated situation of unsynchronised knowledge (30). This situation hinders the efficiency to capture, represent and structure the explicit knowledge in the healthcare domain, making it difficult to set up VRT with good system interoperability (31). Moreover, Burgun et al. (30) any missing knowledge or wrong understanding of the domain can negatively impact the VRT efficiency as alternative training channels. Therefore, it is best to provide explicit knowledge using a well-established mechanism such as ontology.

Fourthly, Zahabi and Razak (8) also mentioned that VRT should also consider user's capabilities, performance and needs to be effective. Often, the development of VRT is majorly focused on fulfilling the technical aspect. When this happens, many VRT users fail to achieve the learning benefits (32). Hence, the design of VRT needs to consider the users' requirements, which can be effectively captured using ontology.

Fifthly, the development of any VR-based training in healthcare must be driven by a well-defined methodology (12). Up till now, there is yet to be any consensus on the best methodology (33) that can be adopted into the healthcare context. The lifecycle of VR development activities needs to be supported with domain knowledge (34) due to the presence of the healthcare's knowledge-intensive tasks and the healthcare's domain complexity involving many stakeholders. So far, according to Gibaud et al. (35), most of the adopted methodologies for the development of VR-related healthcare modules have not been based on well-established methodologies, instead, they have occupied an *ad-hoc* approach. Therefore, it is crucial to find or to have the right methodology to ease the development of VRT in healthcare (36). This can be done by incorporating domain engineering into the lifecycle of the VR methodologies as a way forward.

### Ontology Classification and Roles in Healthcare

**Figure 2:**
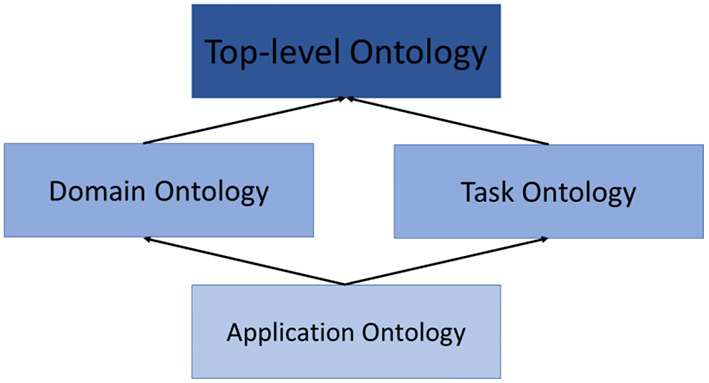
The classification of ontology.

Gruber (37) defined ontology as “a formal and explicit specification of a conceptualization.” In general, there are four types of ontologies, as shown in Figure 2. A top-level ontology is a general-purpose concept that is common across all domains. It is also known as an upper ontology. According to Hoehndorf (38), top-level ontology essentially provides rich definitions that can be applied across multiple domains. Hence, top-level ontology serves as a general foundation for a more elaborated ontology such as domain ontology. Usage of top-level ontology is important as it facilitates reusability, interoperability and much more. Meanwhile, domain ontology is a controlled vocabulary that represents concepts in a specific domain. Task ontology, on the other hand, is a detailed specification that describes the activity-related task. Application ontology describes specific applications.

Other than that, an ontology may also be categorized into a lightweight ontology or a heavyweight ontology. According to Corcho (39), a lightweight ontology is an ontology that describes the relationships between concepts in general associations. Whereas, a heavyweight ontology is an ontology enriched with axioms for semantic interpretation (40). A lightweight ontology is often informal and less expressive as compared to a heavyweight ontology (14).

Another way to classify ontology is based on the ontology motivation, either endurant or perdurant. Colomb and Ahmad (41) define an endurant as an entity that exists timelessly. All of its parts exist at the same time. Meanwhile, a perdurant is an entity that happens in time. If it has parts, it has temporal parts that happen at different times. A domain ontology can be designed to cover endurants and/or perdurants, depending on the domain characteristics and the purpose of ontology to be designed. For example, the domain of interlocking institutional worlds (IWs) such as the one explained by Colomb and Ahmad (41) is perdurant-centric and endurant is considered as a second-class object. Both perdurants and endurants are needed for modeling any kind of domain ontologies under domain IWs characteristics. In short, the endurant-based ontology describes the structural aspect of the domain while the perdurant-based ontology involves the dynamic (process) element of the domain.

#### Role of Ontology in the Development of VRT in Healthcare

The main benefit of using an ontology for VRT in healthcare is to show explicit knowledge in the domain to drive an effective VRT development. A simple example is depicted by (42) who used ontology in biomedicine. The ontology enabled access to domain knowledge, thereby providing a way to verify data consistency and to facilitate integrative analyses over biomedical data.

The healthcare domain is also filled with abundant knowledge that is heterogeneous in nature. Hence, ontology plays a role in providing a structured, common vocabulary that reflects the domain (43). This is important as VRT often involves VR designers and domain experts with different skill competencies and understanding. Hence, ontology can help to bridge the communication gap between the different players of VRT using common (shared) knowledge.

According to Tudorache (32), another main purpose that ontology carries is to enable seamless information sharing and reuse of knowledge between people and software agents, on a domain of interest. This allows the knowledge to be computationally useful and therefore, affects the interoperability of the systems.

To summarize, the role of ontology in the development of VRT in healthcare are:

- depict explicit knowledge in the domain to allow effective VRT development in medical education and training
- enable access to the domain knowledge for verification of data consistency
- provide a structured, common vocabulary that facilitates good systems interoperability
- allow knowledge sharing between different players of VRT

Table 1 demonstrates the characteristics of the existing training in the healthcare domain, the advantages of using virtual reality-based training, and highlights the benefits of applying ontology in VRT development in healthcare.

**Table 1:** Characteristics of existing training in medical education and training, and the potential benefits gained from applying ontologies for VRT development.

**Characteristics of existing training**	**Advantages of using VRT**	**Benefits of applying ontology in VRT**
Restrained by time, sometimes hindered by situations and unavoidable circumstances such as pandemic, emergencies, lack of staff	Allows for training to be done at the time of convenience	Enables sharing and reuse of knowledge to develop other VRTs in medical and education training
Many sub-domains: hence, it is labor-intensive to conduct repeated training	Can be made to allow learning of procedures by other sub-domains	Capture common knowledge that exists across sub-domains
Training is limited to availability, especially when conducted on the real patients	Can simulate any probable situation to which practitioners can act upon	Provides facilitated integrative analyses and validation of data consistency to simulate a virtual training environment
Inadequate infrastructure such as tools to practice (cadavers, sutures, consumables, etc.)	Allows for repeated use of tools to practice (virtual patients, 3D simulated organs, virtual medical tools)	Structure the communication between different players of VRT to provide good system interoperability
Visualization in training is restricted to what the practitioners can see	Enables deeper and more detailed visualizations, up to the molecular level	Capture explicit knowledge in the healthcare domain for effective VRT development
Some training is depended on the patient's consent (in which many patients tend to refuse, such as episiotomy repair)	No consent needed from patients since procedural training is simulated in the virtual environment	Provides facilitated integrative analyses and validation of data consistency to simulate a virtual training environment
Training often comes with a risk to both practitioners and patients (exposure to disease or potential infection)	Minimizes unnecessary risk to both parties	Enable seamless information sharing and reuse

## Methodology

This study reviewed papers focusing on the intersections between virtual reality-based training in the health domain that uses an ontology engineering approach. The initial selection criteria included papers that are journal articles, published from 2015 onwards, and which highlighted the use of ontology in virtual reality-based training in the healthcare domain. However, it was discovered that there are a very low number of papers that met these strict criteria. Hence, the criteria were revised to include:

- Peer-reviewed manuscripts (journal articles, proceedings, books);
- Published from 2010 onwards; AND
- Discussed the use of ontology in virtual reality-based training in the healthcare domain

From the review, we are to reveal in Table 2:

- the classification of the existing ontology in VRT in the healthcare domain;
- the VRT problems that have been addressed using the ontology-driven approach;
- the languages used in the ontology-driven VRT;
- whether the existing ontologies in VRT in the healthcare domain can be reused.

**Table 2:** Overview of the ontology applications for VRT development in the healthcare domain.

**Category of healthcare services**	**Ontology**	**Purpose of ontology**	**Classification of ontology**	**Language**	**Tool**	**Methodology**	**Reuse ontology**	**References**
Medical diagnosis	Ontology of virtual human patient (MV- SYDIME)	Ontology to capture the knowledge of the virtual human patients	Domain, Endurant, Heavyweight	-	Protégé 2000	-	/(SVDIME)	(44)
Medical diagnosis	Ontology for virtual doctor system (VDS)	Ontology to: 1. Explicitly derive knowledge related to all real patients including physical and mental view 2. Identify the right query/ decision based on the previous diagnosis gathered from a professional doctor	Domain, Endurant, Heavyweight	OWL	-	-	×	(45)
Dental treatment	Ontology for dentistry structure	Ontology to provide a semantic description of knowledge and content about the dentistry domain for VRT	Domain, Endurant, Heavyweight	RDF	-	-	×	(46)
Surgery procedures	ONTO-MAMA ontology	Ontology to: 1. To represent domain knowledge related to anatomy part 2. To extend guided surgical training for either students or health professionals in terms of mastering core needle biopsy procedures	Domain, Endurant, Heavyweight	OWL, RDF	Protégé (version 4.1)	Methontology	×	(47)
Dental treatment	Ontology for therapeutic interventions simulation in fixed prosthodontics (VirDent)	Ontology to drive the protocols for preparation of teeth for all-ceramic crowns	Domain, Heavyweight, Endurant	OWL DL, UML	Protégé	Noy and McGuiness	/(DOLCE)	(48)
Rehabilitation/Disease Management	VEULMoR ontology	Ontology to share a common understanding and facilitate the design of a virtual environment	Domain, Heavyweight, Perdurant	OWL, UML	Protégé	Methontology	×	(49)

*(-) not mentioned; (/) yes; (×) no*.

## Ontologies Application in VRT in Healthcare Domain

### Ontology for the Virtual Human Patients (MV-SYDIME)

**Figure 3:**
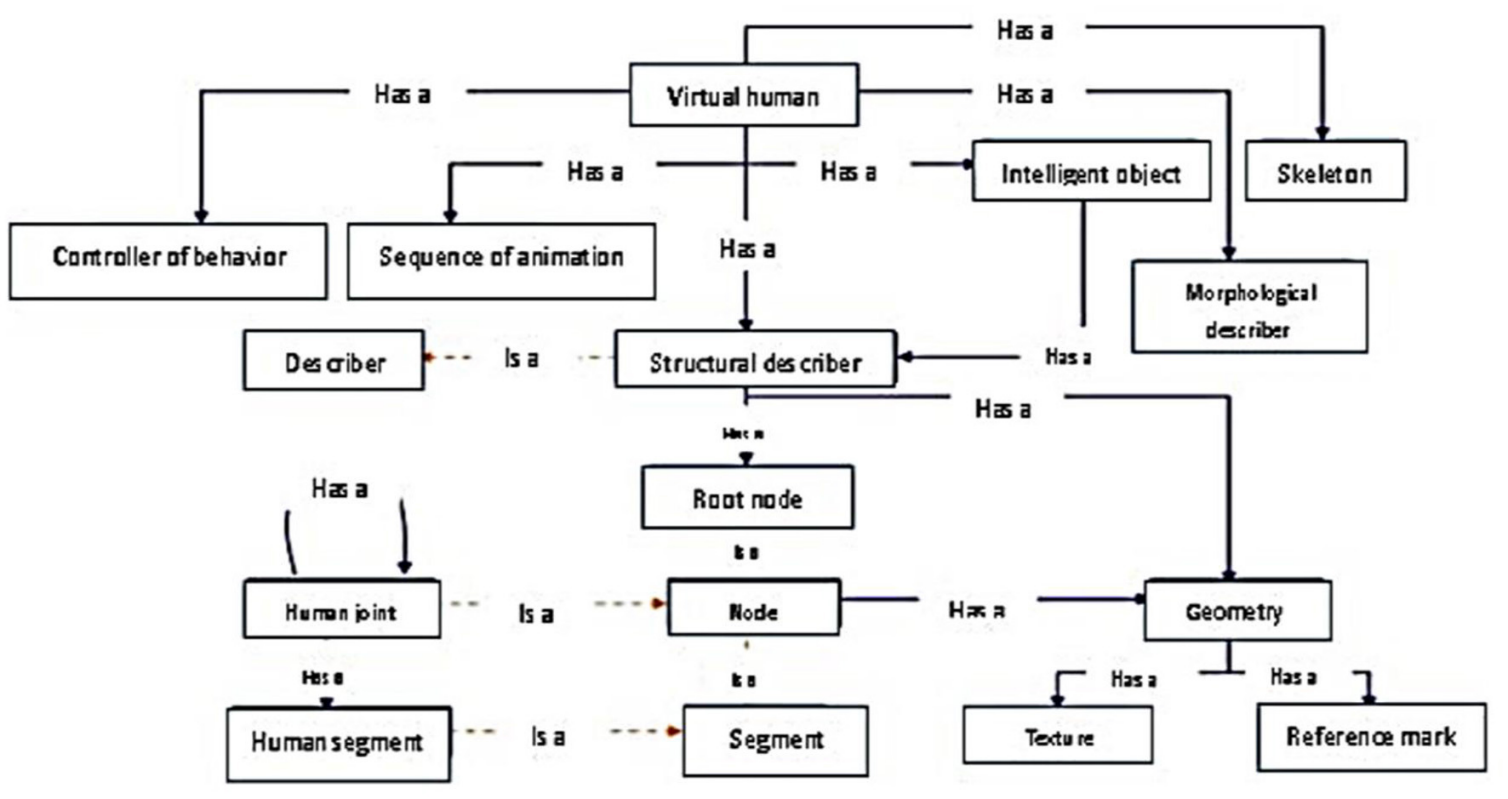
The ontology fragment of virtual human [Source: Monthe et al. (44)].

The lack of experts and inadequate training conditions prevent the progressive learning and training of novice learners in medical diagnostics. This can potentially lead to a higher occurrence of false diagnoses (44). Practicing medical diagnostics is important for learners in healthcare to confidently decide, confirm and explain their diagnosis. Hence, this paper discussed developing a virtual patient. However, the human body is very complex to model entirely. Hence, in this study, they used MV-SYDIME ontology to capture the knowledge of the virtual human patients and represent the pathological concepts (see Figure 3) to ensure good interoperability among the systems involved.

### Ontology for Virtual Doctor System (VDS)

**Figure 4:**
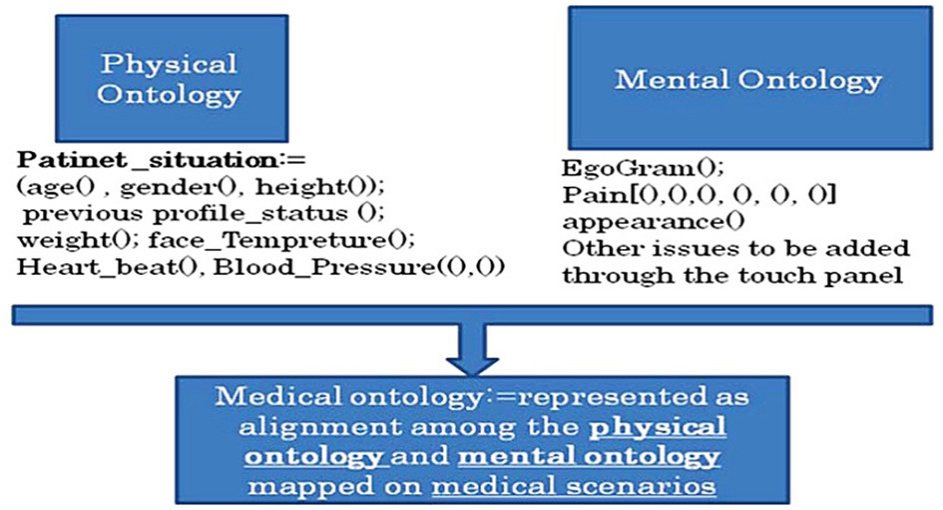
The ontology fragment of virtual doctor system [Source: Fujita et al. (45)].

Patients usually wait for hours to see the doctor. Hence, this study focused on designing an avatar that resembles a real human doctor to interact with the patients. This avatar, or the virtual doctor, will perform the initial diagnosis to classify patients based on how critical their cases are. The nature of the knowledge that exists in this kind of situation is not only abundant but also heterogeneous. To effectively deal with this, Fujita et al. (45) used ontology to build the probabilistic model of the VDS system. The model can perform medical diagnosis based on the doctor's knowledge and experience and then, sort the patient cases according to the generated weight (high, medium, low, not-simple etc.). Two types of ontologies, Physical Ontology (PhO) and Mental Ontology (MeO) are aligned and represented on Medical Ontology as shown in Figure 4. Both ontologies are independent of each other. The medical ontology represents the conceptual view of the medical diagnosis and the specialization is according to the doctor's experiences.

### Ontology for Dentistry Structure

**Figure 5:**
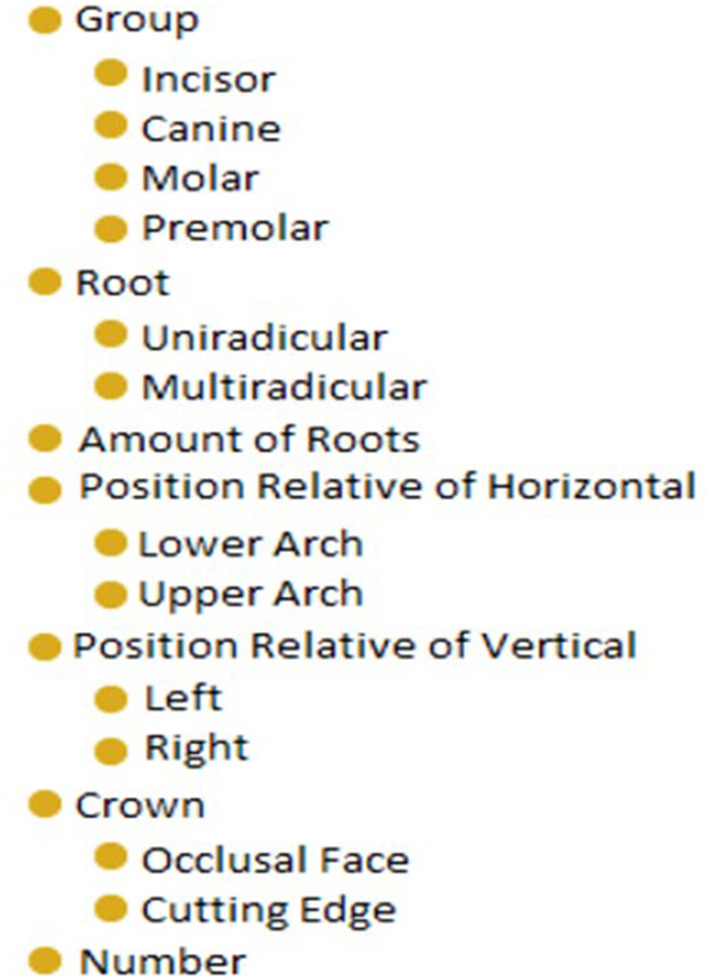
The ontology fragment of dentistry structure [Source: Dias et al. (46)].

Dentistry anatomy is an area of important knowledge in which a dentist needs to be very well-versed. Hence, a study by Dias et al. (46) utilized virtual reality-based training to elevate the visualization of a dental structure as part of the learning process. The advanced VR system incorporates ontology as shown in Figure 5, to provide a semantic description of knowledge to the virtual 3D dental structures. Endurant ontology was used in the development process. However, the authors' paper did not mention the used tool and methodology for designing their ontology, other than indicating the use of an RDF file. This language enabled the semantic description and aggregation of multimedia contents as a 3D model. The VRT was evaluated by ten professionals in dentistry, to which all users believed that the system can be used as a training tool to support teaching dentistry structure and content.

### Ontology and 3D Graphic Model of the Female Breast Anatomy (OntoMAMA)

**Figure 6:**
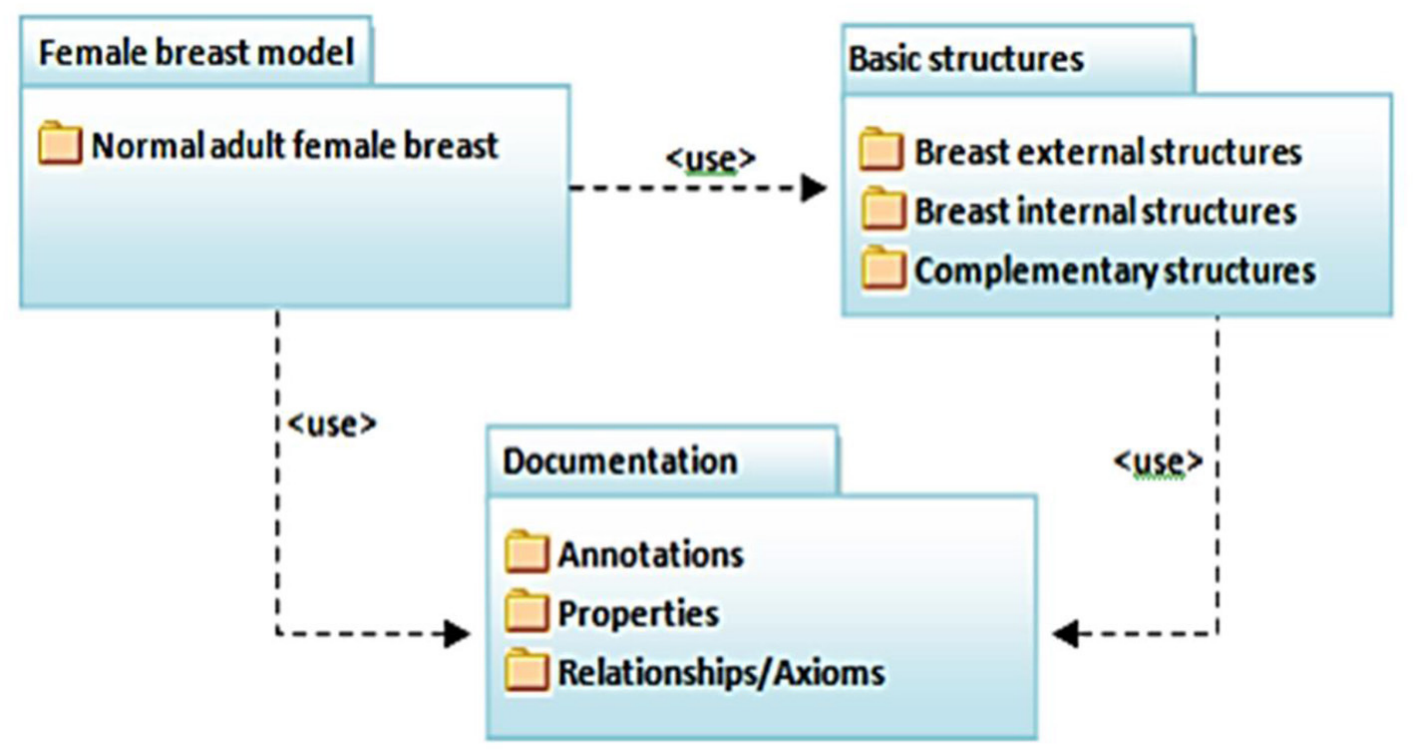
The ontology fragment of ONTO-MAMA [Source: Klavdianos et al. (47)].

Artificial intelligence and virtual reality have shown their potential to be used in medical training and simulations. Yet, organizing the knowledge of complex human anatomy and complicated medical procedures requires well-known mechanisms as the main guidance to develop good VRT modules. This study discussed the use of an ontology to express the vocabulary of the female breast anatomy in a virtual reality environment. As depicted in Figure 6, ONTO-MAMA ontology is a useful reference to provide guided surgical training for trainees to master core needle biopsy procedures. Klavdianos et al. (47) used the Methontology method and the Protégé tool in ONTO-MAMA ontology design. The OWL language was chosen to represent the domain knowledge related to the selected anatomy parts.

### Ontology for Therapeutic Interventions Simulation in Fixed Prosthodontics (VirDent)

**Figure 7:**
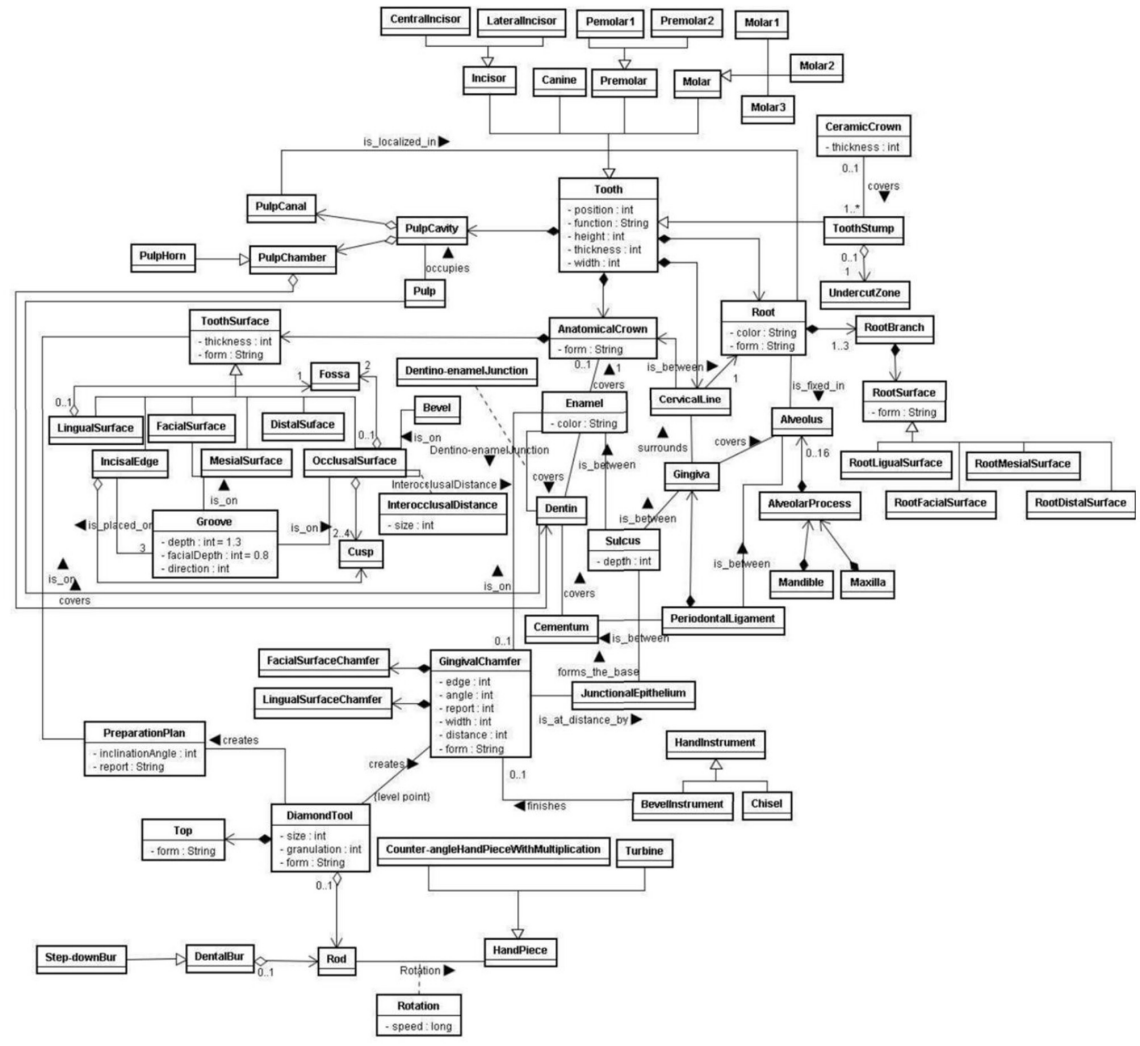
The ontology fragment of VirDent [Source: Klavdianos et al. (47)].

Prosthodontics is one branch of dentistry that deals with the restoration of missing teeth using prostheses. One of the procedures in prosthodontics includes teeth crowning. Teeth crowning comes with many restoration options; however, all-ceramic restoration is one of the most biocompatible restorations available. Nonetheless, it is not widely used because the procedure is delicate and requires high precision skills. Any misstep in all-ceramic restoration may lead to adverse effects such as pulp inflammation. In 2011, Bogdan described e-learning, a virtual reality-based system called VirDent. The sole purpose of the VirDent system is to help dentistry students learn how to prepare fixed teeth prosthodontics for ceramic crowns. To formally construct a knowledge base and synchronize the knowledge for the VirDent system architecture, a domain ontology is established using the OWL language and Protégé tools (see Figure 7).

### VEULMoR Ontology

**Figure 8:**
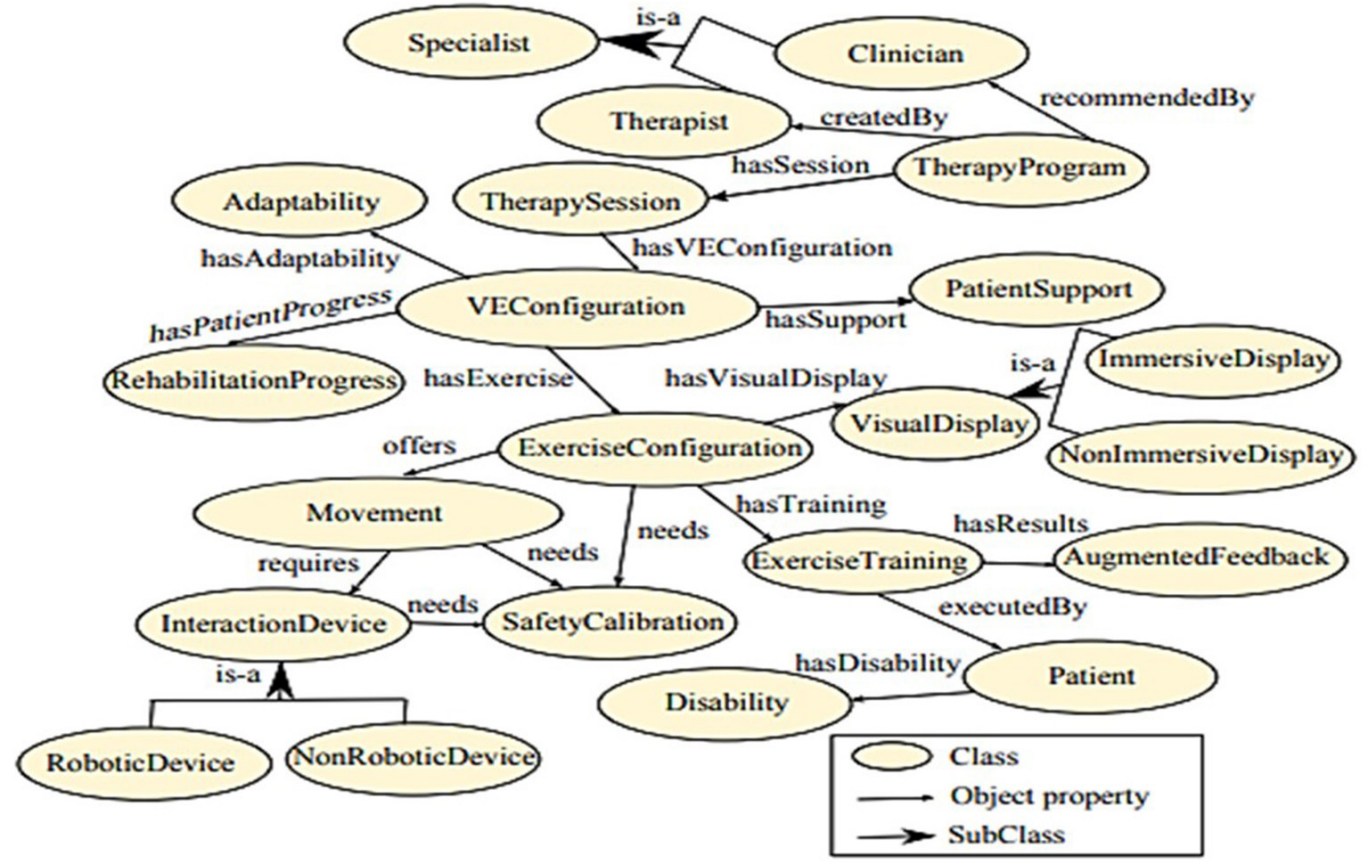
The ontology fragment of VEULMoR [Source: Ramírez-Fernández et al. (49)].

Even as VRT began to expand to many subdomains of healthcare, the design of VR-based training remains a trivial task. A study by Ramírez-Fernández et al. (49) focused on the issues faced in designing VR for upper limb motor rehabilitation. They argued that the available approaches to designing VR are insufficient, as no considerations are made to understand the knowledge in the domain, hence it is difficult to maintain a standardized vocabulary. A simpler mechanism to capture all this knowledge and represent it in a common language is required. Ramírez-Fernández et al. (49) designed the VEULMoR ontology (see Figure 8) to make it easier to design a virtual environment for upper limb motor rehabilitation using VR. The ontology can capture distinct aspects of the domain such as stroke-survivor, characteristic motor rehabilitation, interaction devices, and others.

## Discussion

As depicted in section Methodology, VRT development in healthcare that occupies the ontology engineering approach is not discussed extensively in the available scholarly literature. It indirectly suggests that the potential of using ontology in VRT development has not been fully explored nor given much attention.

From the overview, it can be learned that the ontology was mainly used to represent the common knowledge in the healthcare domain; however, each type of knowledge is very specific and suits specialized VRTs. However, the ontology is sufficient to bridge the communication problems between the SMEs (subject matter experts) and the technology experts (VR developer, 3D designer, etc).

Most of the ontology classification in healthcare VRTs can be narrowed down to domain ontology, as the main purpose is to explicitly capture the domain knowledge. Additionally, the existing ontology mostly falls under the heavyweight ontology category. This is consistent with the need for VRTs in healthcare, which requires formal standardization and expressiveness. However, heavyweight ontology uses lots of computational power and memory, hence utilizing considerable resources for understanding the specifications and maintaining the interoperability of the system (14). Sometimes, this becomes the limiting factor that deters further growth of VRT in healthcare.

Additionally, perdurant knowledge is not heavily considered in many ontology designs for VRT in healthcare. As shown in Table 2, many ontology developments in the healthcare domain tend to favor object-oriented ontologies (structural aspect), also known as endurant ontologies. Endurant knowledge represents the structural elements within the domain, while perdurant knowledge involves the dynamic elements of the domain. Since the healthcare domain is often flexible and dynamic, the perdurant design of ontology should not be neglected.

The language used for ontology design in many VRTs in healthcare is mostly OWL. Generally, OWL can be quite intimidating for non-expert users as it has a steep learning curve (50). To add to this, the current ontology editors, such as Protégé, offer an intimidating interface for ontology development. Therefore, to encourage the adoption of ontologies in VRT development in the healthcare domain, the ontology design needs to be enhanced with developer-friendly tools and languages such as the UML-based ones.

The overview also denoted that a majority of the research did not disclose or explain the methodology used for the ontology design for VRTs in healthcare. Only two researchers mentioned the use of Methontology (47, 49), and another research by Bogdan (48) referred to the basic ontology development guideline (51). Additionally, the methodology in the literature also failed to discuss how the ontology engineering methodologies are blended or incorporated into VR methodology development. We argue that for healthcare purposes and context, establishing a generic ontology development methodology tailored to the domain-specific context is worthy since most ontology development methodologies are not designed for the general purpose of the domain (35). Moreover, there is also no consensus on the best ontology development methodologies so far (33).

Nonetheless, this carved the path toward having more research opportunities that would be able to establish on integrating or incorporating ontologies into VR development methodologies. For example, using ontology to design virtual environment for usability (52), interfaces (53) and applications (54).

The overview also depicted that ontology reuse across a given domain is not a consolidated practice. Enacting reuse of ontology in practice is difficult due to the heterogeneity in the conceptualization, the difficulty to select the suitable ontology to reuse, the struggle to extract the subset of ontology to reuse and maintaining the extracted subset as the source to which the ontology evolves. Yet, reusability is one of the important characteristics of a good ontology. Reusability of the ontology can drastically minimize the time and labor needed for building up new learning scenario models. As a result, the ontology promisingly speeds up the development of VRT processes while reducing the cost at the same time. Hence, the current situation needs to adopt advanced usage of the ontology engineering approach, such as the utilization of upper-level ontologies that can govern reusability.

The importance of a well-designed ontology for VRT can easily be explained using a “map” analogy. Ontology captures all the knowledge within a map. A world map contains all the knowledge of the “world.” Yet, it is only meaningful if it is used for a certain interest or purpose. For example, the map in the Waze application, the map in navigation systems or the map in any GIS application uses the same map, but the interest and purpose are different. Hence, ontology in VRT is important to capture the whole knowledge within the domain to facilitate the different uses and to make it meaningful. So far, we have identified the limitations to the current ontology design for VRTs in healthcare through this overview. To have an ontology for VRT that can be utilized on the same page, we need a common framework that can govern its development and its utilization purpose. Therefore, an ontology engineering approach such as adopting the right upper ontology system can play an important role. Choosing the right ontology language that enables two-way transformation (e.g., OWL-UML vice versa) and is familiar to all stakeholders is also essential.

Nevertheless, for each existing VRT, ontology has shown huge potential in capturing, representing and sharing the knowledge within a complex domain such as healthcare. However, most of the existing ontologies are used in the implementation phases of VRT development life cycles. This causes the role of ontology to be insufficient. An ontology needs to be integrated earlier in the VRT development life cycle. Only then, the ontology can seamlessly share the common knowledge between the people and the system. Without an ontology, the resulting VRTs are often poor with a lack of relatedness, immersiveness, and being user-centered.

**Figure 9:**
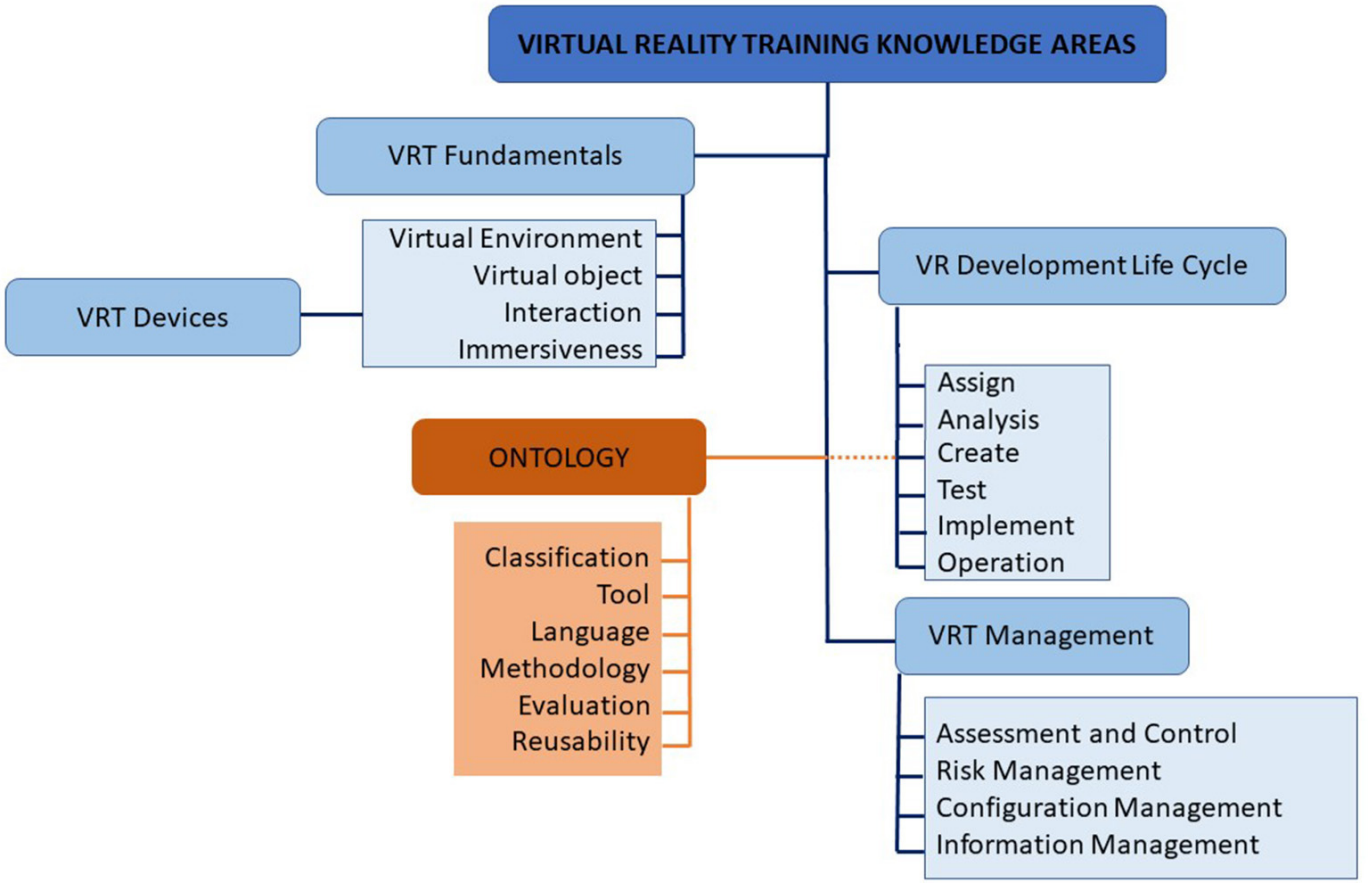
Proposed linkage of ontology and base of knowledge for VRT development in healthcare.

Healthcare is a knowledge-intensive domain. And many aspects of healthcare need explicit knowledge. Therefore, when we want to transfer this knowledge into the VR world, there is a need for a clear reference on these knowledge structures and controlled vocabularies. In this sense, an ontology can become the reference model. However, looking at the current progress of ontology for VRT development, it is not based on well-designed ontologies. Therefore, in Figure 9, we proposed a base of knowledge for VRT in healthcare to assist the VRT development in healthcare through an ontological approach.

The bases of knowledge for VRT in healthcare are divided into four, which are, VRT fundamentals, VRT devices, VRT development life cycle, and VRT management. In the scope of VRT for healthcare, the training models often involve interactions between virtual objects and virtual environments to achieve immersiveness and are often aided by the relevant VR devices. VRT development life cycles involve six phases, which are assignment, analysis, creation, testing, implementation, and operation. The existing ontologies are mostly integrated into the later phases of VRT development.

To increase the adoption of an ontology, consideration to involve the use of the ontology in the earlier phases is recommended. This can be done through the involvement of the domain experts in different stages of the VRT development process, for them to provide the necessary inputs and feedback (55).

Additionally, having ontology in VRT development will mitigate for better VRT management. This includes the management of assessment, control, risk, configuration, and information. An ontology represents the common knowledge that is applicable and understood by both technical developers and domain experts, as well as ease of systems interoperability.

The healthcare domain is complex due to the massive “knowledge-intensive” tasks. The training, in the traditional sense, involves many procedures, regulations, rules, and processes that need to be followed strictly. Therefore, translating these traditional training aspects into VR-based training can be quite a tedious task. Looking at the possibility whereby every single procedure needs to be converted into a VR-based procedure (regardless of being semi or fully immersive), we need to have explicit knowledge and its intended meaning to realize the possibilities. All the knowledge must be well-represented, accurate and applicable to the intended community. Without an ontology mechanism, it will not be easy to support VRT development in healthcare.

Another missing aspect in the current VRTs in the healthcare domain is the lack of discussion on how far the learning theories or educational models have been integrated to support the development of VRT. The overview depicted little evidence on any theories being applied for the development of the VRTs in healthcare. This is in agreement with a study by Zhou et al. (16). They suggested that the design of VR-based educational applications should also consider pedagogical models, apart from focusing on the technical perspectives of VR, to achieve the learning benefits.

Chimalakonda and Nori (56) also argued that most educational technologies today lack the support of strong instructional design knowledge. Yet, this limitation can easily be addressed through the use of ontology. Ontology can help decode the knowledge of the domain, represent it explicitly, and make the knowledge interpretable for machine processing. This will eventually open more doors of opportunity for further exploration and expansion of VRT in healthcare.

## Conclusion

This review contributes to a holistic examination of the primary studies relevant to the topic of ontology-engineering in virtual reality-based training in healthcare, spanning the last decade. The findings provide a comprehensive understanding of and shed new lights on (1) the existing state of VRT development in healthcare supported by ontologies, (2) the contribution that ontologies make to the current VRTs in healthcare, (3) the state of ontology design for VRT development in healthcare, (4) and the limitations to the available ontologies in terms of reusability and future expansion of VRT in healthcare. From the overview, the adoption of ontology in the development of VRTs in healthcare is still at an infancy stage. This is based on the lack of scientific research and through analysis and literature on how ontology could be used in the development process of VRT in a variety of healthcare disciplines.

Despite the low number of scholarly papers discussing ontological perspectives of VRT in healthcare, the ontology engineering approach has begun to garner a great deal of attention, as it is one of the most pertinent mechanisms that can address the issues that deter the VRTs development in healthcare. Moreover, the reusability of the existing ontologies is low, to map concepts to concepts within the VRTs in healthcare. A comprehensive strategy to create an effective ontology for VRT in healthcare may include (1) tackling the base of knowledge for VRT in healthcare, (2) improving the ontology design through consideration of requirements from various stakeholders and adopting advanced ontology design principles such as the upper ontologies system, (3) identifying well-defined learning theories or models to strengthen the foundations, and (4) becoming part of the solution to VRT limitations.

There are two important bodies of knowledge (BOK) that this paper can spark further directions in, in terms of research opportunities. Firstly, how can we improve existing ontologies for supporting VRT from the lens of ontology engineering? By answering this question, the researcher would be able to make contributions to the BOK of ontology engineering, BOK-OE in short. For instance, new knowledge can be added to the BOK-OE such as a new approach on ontology design, new guidelines, new strategy, new knowledge from lessons learned in the healthcare domain context, new ontology development methodologies, and more. Secondly, how fundamental, or practical problems in BOK-VRT can be solved using ontology-based solutions? By answering this question, the researcher would be able to make contributions to the BOK-VRT. For example, fundamental issues concerning VRT management and evolution (see Figure 9) can be resolved by having an ontological approach as a solution.

## Author Contributions

MA contributed to the conception and design of the study. UM wrote the first draft of the manuscript. AS enhanced the VR-related sections of the manuscript while MB contributed to the sections with regard to healthcare virtual trainings. All authors contributed to manuscript revision, read, and approved the submitted version.

## Conflict of Interest

The authors declare that the research was conducted in the absence of any commercial or financial relationships that could be construed as a potential conflict of interest.
